# Effect of cell separation on gene expression and DNA methylation profiles in intestinal epithelial cells

**DOI:** 10.1186/1756-8935-6-S1-P32

**Published:** 2013-03-18

**Authors:** M Molitor, J Postberg, V Orth, M Zilbauer, AC Jenke

**Affiliations:** 1 Department of Neonatology, HELIOS Children’s Hospital, Witten/Herdecke University, Germany; 2 Department of Paediatric Gastroenterology, University of Cambridge, Addenbrooke’s Hospital, UK

## 

Epigenetic signatures are highly cell type specific. Separation of distinct cell populations is therefore a prerequisite for all epigenetic studies. To date little information is available whether separation protocols might influence epigenetic and/or geneexpression signatures. We therefore investigated the influence of two frequently used protocols to isolate intestinal epithelium cells (lECs) – EDTA/DTT and enzymatic release –on gene expression and DNA methylation patterns of lECs obtained from 6 healthy individuals. While cell purity was >95% using both approaches, gene expression analysis revealed significantly higher mRNA levels of several inflammatory genes in EDTA/DTT when compared to enzymatically released cells. In contrast, DNA methylation of selected genes was less variable but tended to be lower in inflammatory and higher in maintenance genes in EDTA/DTT released cells. Taken together, this highlights the importance of considering the effect of cell separation procedures particularly when it comes to correlating epigenetic changes with gene expression.

